# Fibroblast activation protein-based theranostics in cancer research: A state-of-the-art review

**DOI:** 10.7150/thno.69475

**Published:** 2022-01-09

**Authors:** Liang Zhao, Jianhao Chen, Yizhen Pang, Kaili Fu, Qihang Shang, Hua Wu, Long Sun, Qin Lin, Haojun Chen

**Affiliations:** 1 Department of Nuclear Medicine and Minnan PET Center, The First Affiliated Hospital of Xiamen University, Xiamen, China; 2 Department of Radiation Oncology, The First Affiliated Hospital of Xiamen University, Xiamen, China; 3 Department of Oncology, The Second Affiliated Hospital of Jiaxing University, Jiaxing, China

**Keywords:** cancer-associated fibroblasts (CAF), fibroblast activation protein (FAP), targeted radionuclide therapy, PET/CT, cancer management

## Abstract

In recent years, quinoline-based fibroblast activation protein (FAP) inhibitors (FAPI) have shown promising results in the diagnosis of cancer and several other diseases, making them the hotspot of much productive research. This review summarizes the literature for the state-of-the-art FAPI-PET imaging for cancer diagnosis compared with fluorodeoxyglucose (FDG)-PET. We also summarize the use of FAPI-PET for therapeutic regimen improvement and fibroblast activation protein (FAP)-targeted molecule modification strategies, as well as preliminary clinical studies regarding FAP-targeted radionuclide therapy. Our qualitative summary of the literature to date can inform future research directions, medical guidelines, and optimal clinical decision-making.

## Introduction

Approximately 19.3 million new cancer diagnoses and approximately 10.0 million cancer deaths occurred worldwide in 2020, according to estimates from Global Cancer Statistics 1. Cancers develop in complex environments composed of tumor cells and the surrounding stroma. The “seed and soil” theory emphasized the interactive importance of both components as early as 1889 2. However, diagnostic and therapeutic paradigms have predominantly targeted only tumor cells.

The widespread application of immunotherapy in clinical trials has led to increased research attention being paid to the tumor microenvironment (TME). The tumor stroma or TME comprises all the noncancer components in the tumor tissue, including cancer-associated fibroblasts (CAFs), the extracellular matrix (ECM), various types of immune cells, and intertwined blood vessels. The TME can develop an immunosuppressive niche in which tumor cells are protected from conventional therapies, resulting in treatment failure 3.

CAFs are among the most abundant components of the TME in solid tumors 4. However, CAFs are heterogeneous cells with both tumor-promoting and tumor-suppressive effects observed in different situations 5. CAFs model and remodel the ECM structure, which can become a physical barrier against the infiltration of immunocytes, which have a killer function, or a structural scaffold for intercellular interaction between tumor cells and non-tumor cells in the TME, thereby regulating tumor initiation, neovascularization, and metastasis 6. On the other hand, CAFs can secrete multiple chemokines and cytokines, such as transforming growth factor-β (TGFβ), CC-chemokine ligand 2 (CCL2), and interleukin-6 (IL-6), in order to recruit immunocytes with inhibitory functions in the tumor stroma, thereby facilitating immune evasion 7.

CAFs have several biological markers, including α-smooth muscle actin, fibroblast activation protein (FAP), and platelet-derived growth factor receptor-β (PDGFRβ). FAP is a type II integral membrane glycoprotein belonging to the serine protease family involved in ECM remodeling and fibrogenesis 8. In colorectal cancers, FAP expression is higher at the invasive front than in the tumor center 9.

Although the prognostic value of FAP in cancers has been inconsistent throughout the literature, high expression of FAP has been shown to be an independent poor prognostic marker for outcomes in lung cancer, hepatocellular carcinoma, and colon cancer in studies with large sample sizes (n = 138-449 patients) 10-12. In a murine model, tumor growth could be potentiated by the constitutive expression of FAP, which could, in turn, be meaningfully attenuated by anti-FAP antibodies 13. However, sibrotuzumab (a humanized version of the murine anti-FAP antibody) failed as a treatment regimen in an early phase II trial for metastatic colorectal cancer 14. Although FAP antibodies have shown limited response in tumor therapy, small molecules targeting FAP have attracted increasing attention in the area of tumor theranostics.

## Targeting FAP for tumor imaging

FAP, expressed at low levels in healthy tissues, is involved in various pathological conditions and is detected in over 90% of malignant epithelial tumors 8, 15. Thus, molecular imaging (including positron emission tomography [PET] and single-photon emission computed tomography [SPECT]) targeting FAP is a promising diagnostic imaging modality.

A series of quinoline-based FAP inhibitors (FAPIs) was developed by the University Hospital Heidelberg group based on clinical and preclinical research 16-18. The first FAPI variants (FAPI-01 and FAPI-02) were reported in 2018, demonstrating that FAPI-02 has an improved binding affinity to human FAP compared with FAPI-01 16. Impressively, ^68^Ga-FAPI-02 PET images showed favorable tracer uptake in tumor tissues and low background uptake in normal healthy organs, resulting in high-contrast images in tumor-bearing murine models and three cancer patients 16. Subsequently, FAPI-04 was identified as the best tracer from among 15 modified FAPIs for PET imaging applications, showing a higher tumor uptake in murine xenograft models than FAPI-02 17. Notably, ^68^Ga-FAPI-04 was evaluated in a cohort of 80 patients presenting with 28 types of tumors (54 primary tumors and 229 metastases). It showed overall intense tracer uptake (with a maximum standard unit value [SUVmax] > 6) and high-contrast images in various highly prevalent cancers, including sarcomas, cholangiocarcinoma, and esophageal, breast, lung, hepatocellular, colorectal, head-neck, ovarian, pancreatic, and prostate cancers 19. To improve the pharmacokinetics of this PET tracer, FAPI-46 was discovered from 15 other FAPI derivatives, and it demonstrated an enhanced tumor-to-background ratio (TBR) compared with FAPI-04 18. Interestingly, ^68^Ga-FAPI-46 PET/CT imaging acquisition at an early time point (10 min p.i.) had an equivalent lesion detection rate compared with a late time point (60 min p.i.) in a previous study 20.

Since the dodecane tetraacetic acid (DOTA) ligand was used as a chelator, FAPI-04/46 could also be radiolabeled with therapeutic nuclides such as ^90^Y, ^177^Lu, and ^225^Ac. In addition, the NOTA ligand was used in FAPI-74 for labeling with both ^18^F and ^68^Ga, showing favorable image contrasts in PET/CT imaging in various cancers 21. Similarly, the chelator bis((1-(2-(tert-butoxy)-2-oxoethyl)1H-imidazol-2-yl) methyl) glycine was applied to [^99m^Tc]Tc-Labeled FAPI tracers, and [^99m^Tc]Tc-FAPI-34 SPECT was performed on two patients with ovarian and pancreatic cancers 22.

It should be noted that increased FAPI uptake has been reported in many non-oncological conditions, including inflammatory lesions, fibrotic disease, trauma, arthritis, degenerative bone disease, immunoglobulin 4 [IgG-4] related diseases, connective tissue disease, and atherosclerosis 23, 24. Thus, imaging interpretation with ^68^Ga/^18^F-FAPI PET/CT should be interpreted with caution to avoid misdiagnosis. Interestingly, increased FAPI uptake in non-oncological conditions could open possibilities for broader use of FAPI for corresponding diseases. For example, FAP-specific PET/CT could be used in the discrimination between inflammatory and fibrotic activity in IgG-4 related diseases 25, evaluation of the progression in atherosclerotic plaques 26, and assessment of the disease activity in fibrotic interstitial lung diseases 27, 28.

## Comparing ^68^Ga-FAPI and ^18^F-FDG uptake in various types of cancer

**Figure 1:**
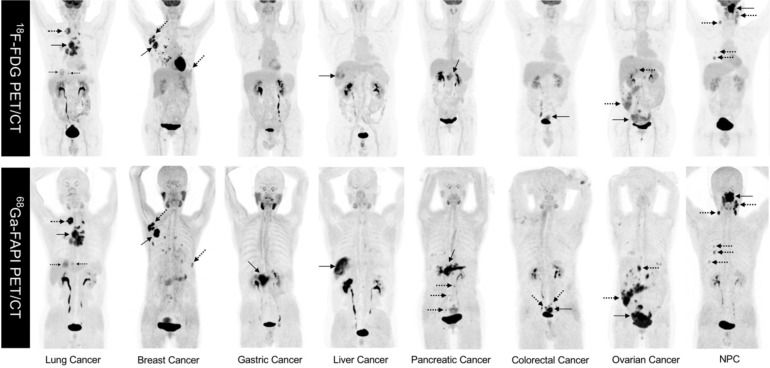
Representative comparison of 8 patients with different tumor entities undergoing both ^18^F-FDG PET and ^68^Ga-FAPI-04 PET imaging within less than 1 week. Solid arrows indicate primary tumors, while the dotted arrows indicate metastasis lesions. NPC: nasopharyngeal carcinoma.

As FAPI is a novel PET tracer in cancer imaging, it is critical to evaluate lesion detection rates and diagnostic efficacy for FAPI compared with ^18^F-fluorodeoxyglucose (^18^F-FDG), the dominant tracer in oncology. To the best of our knowledge, Chen *et al.* conducted the first head-to-head study comparing ^68^Ga-FAPI-04 and ^18^F-FDG PET/CT in a cohort of 75 patients (54 patients identified at initial assessment and 21 patients with recurrence detection) with 12 different tumor entities. This prospective study demonstrated that ^68^Ga-FAPI-04 had a higher sensitivity as compared with ^18^F-FDG in identifying primary tumors (98.2% *vs.* 82.1%, P = 0.021), lymph node metastases (86.4% *vs.* 45.5%, P = 0.004), and bone and visceral metastases (83.8% *vs.* 59.5%, P = 0.004) 29. However, the limited number of patients harboring each cancer type enrolled in this study did not allow for a subgroup comparison in terms of diagnostic efficacy for the same tumor type. Representative MIP images of both ^18^-FDG PET/CT and ^68^Ga-FAPI PET/CT in 8 patients with different types of cancer are shown in Figure 1. Some studies have recently compared the diagnostic efficacy of ^68^Ga-FAPI and 18F-FDG in various types of tumors.

### Head and neck cancer

^68^Ga-FAPI-04 PET/CT demonstrated improved sensitivity when compared with ^18^F-FDG PET/CT for cancers of unknown primary origin (CUP), while the sensitivity for detecting primary tumors was comparable in nasopharyngeal carcinoma (NPC), oral squamous cell carcinoma, and Waldeyer's tonsillar ring cancer 30-34. Impressively, ^68^Ga-FAPI-04 PET/CT pinpointed 39% (7/18) of primary head and neck CUP tumors among primary patients with negative ^18^F-FDG findings 30. In another cohort of 45 patients with NPC, ^68^Ga-FAPI-04 PET/CT showed higher radiotracer uptake than ^18^F-FDG for primary tumors, regional lymph nodes, and distant metastases, resulting in higher sensitivity for the detection of lymph nodes and distant metastases 31. Interestingly, Qin *et al.* reported that ^68^Ga-FAPI-04 PET/CT detected a smaller number of positive lymph nodes compared with ^18^F-FDG PET/CT (the detected number of positive lymph nodes was 48 *vs.* 100) 32. However, no suspicious FDG-avid lymph nodes were confirmed histologically. This study indicated that ^68^Ga-FAPI PET/CT might be more specific than ^18^F-FDG for differentiating reactive lymph nodes from tumor metastatic lymph nodes 35, and it is very likely that the FDG-positive/FAPI-negative lymph nodes are reactive lymph nodes. However, this finding requires validation in future research.

Both studies mentioned above demonstrated that ^68^Ga-FAPI outperformed ^18^F-FDG PET/CT in evaluating skull base and intracranial invasion in NPC 31, 32. In a study of 10 patients with oral squamous cell carcinoma (OSCC), both ^68^Ga-FAPI-04 PET/CT and ^18^F-FDG PET/CT had comparable sensitivity and specificity for detecting primary tumors (100% *vs.* 100%) and cervical lymph node metastases (81.3% *vs.* 87.5%, P = 0.32; 93.3% *vs.* 81.3%, P = 0.16) 33. However, both radiotracers have relatively high physiological uptakes in the oral mucosa, potentially leading to a compromised target-to-blood pool ratio (TBR) 33. For cancers of Waldeyer's tonsillar ring, ^68^Ga-FAPI-04 PET/CT showed a higher detection rate in primary tumors but an inferior detection rate in lymph node metastases compared with ^18^F-FDG PET/CT. However, ^68^Ga-FAPI-04 PET/CT demonstrated a higher TBR than ^18^F-FDG PET/CT for detecting primary tumors (10.90 *vs.* 4.11) 34.

### Digestive system cancer

^68^Ga-FAPI-04 PET/CT demonstrated improved sensitivity in liver, gastric, and pancreatic cancers when compared with ^18^F-FDG PET/CT 36-45, while the sensitivities of both tracers were comparable in colorectal and esophageal cancers 39, 46. For liver cancer, including hepatocellular carcinoma and intrahepatic cholangiocarcinoma, ^68^Ga-FAPI-04 has been demonstrated to have a higher sensitivity in detecting primary liver tumors (partly attributed to higher tumor uptake and lower hepatic background uptake as compared with ^18^F-FDG) as well as extrahepatic metastases 36-38. In gastric cancer, the sensitivity of ^68^Ga-FAPI-04 PET/CT was higher than that of ^18^F-FDG PET/CT in detecting primary tumors, lymph nodes, and distant metastases 39-42, 45, especially for signet-ring cell carcinoma and mucinous adenocarcinoma.

In gastric cancer, Pang *et al.* and Qin *et al.* reported that the FAPI-derived SUVmax in primary and metastatic lesions was significantly higher than the FDG-derived SUVmax 39, 40, while Jiang *et al.* and Kuten *et al.* reported that FAPI-derived TBR was higher than FDG-derived TBR without accompanying differences in the SUVmax 41, 42. In colorectal cancer, ^68^Ga-FAPI-04 PET/CT has been demonstrated to have equal sensitivity in primary tumor detection compared to ^18^F-FDG PET/CT (6/6, 100%), although the FAPI-derived SUVmax was statistically significantly higher than the FDG-derived SUVmax in a previous study 39.

Although ^68^Ga-FAPI-04 has significantly higher uptake compared with ^18^F-FDG in esophageal cancer, their sensitivity in detecting primary tumors was shown to be comparable 46. Pancreatic tumors are characterized by intense stromal desmoplastic reactions surrounding cancer cells, and CAFs are the main effector cells involved in this desmoplastic reaction. As expected, ^68^Ga-FAPI-04 PET/CT shows higher sensitivity in detecting primary tumors, including lymph nodes, and metastases than ^18^F-FDG PET/CT in pancreatic cancer, mainly due to the intense ^68^Ga-FAPI uptake in pancreatic tumor lesions 43. However, non-specific ^68^Ga-FAPI uptake in tumor-induced pancreatitis should be noted, as intense ^68^Ga-FAPI uptake is normally observed throughout the whole pancreas in this disease. This phenomenon is frequently observed in tumors located in the head of the pancreas. As a solution, dual-time point ^68^Ga-FAPI PET/CT (1 h early-point and 3 h late-point scans) may help differentiate pancreatitis from malignancy 43, 44.

### Breast cancer

^68^Ga-FAPI-04 PET/CT was demonstrated to detect a greater number of cancerous lesions with a higher SUVmax for primary tumors, including lymph nodes, and distant metastases compared with ^18^F-FDG PET/CT in a cohort of 48 breast cancer patients 47. Kömek *et al.* reported a similar conclusion in a cohort of 20 patients 48.

### Lung Adenocarcinoma

Both ^18^F-FAPI-42 and ^18^F-FDG had comparable detection rates (100%) for primary tumors in a cohort of 34 patients 49. Moreover, ^18^F-FAPI-42 showed higher SUVmax compared to ^18^F-FDG in the lymph nodes, pleura, bones, and other tissue lesions (P < 0.05), as well as better TBR compared to ^18^F-FDG in brain lesions (9.53 ± 12.07 *vs*. 1.01 ± 0.49, P < 0.0001). Therefore, ^18^F-FAPI-42 may have advantages over ^18^F-FDG for the primary staging of lung adenocarcinoma. However, ^18^F-FAPI-42 is inferior in detecting brain lesions compared to contrast-enhanced magnetic resonance imaging (CE-MRI) (56 *vs*. 34, P = 0.002). Interestingly, one recent study demonstrated intense ^68^Ga-FAPI activity but minimal ^18^F-FDG uptake in a malignant pulmonary ground-glass opacity (GGO) nodule 50. Further studies are needed to determine the effectiveness of ^68^Ga-FAPI and ^18^F-FDG PET/CT for characterizing GGO nodules.

### Sarcomas

The detection rates for primary lesions observed with ^68^Ga-FAPI-46 PET/CT and ^18^F-FDG PET/CT were equally high (33/43 *vs.* 35/43) for bone or soft tissue sarcomas. Moreover,^ 68^Ga-FAPI-46 PET/CT allowed for detecting additional distant metastatic lesions that were not detected via ^18^F-FDG PET/CT in 6/43 patients 51.

### Hematological neoplasms

Head-to-head comparison studies of hematological neoplasms are relatively rare compared to studies evaluating solid tumors. Lan *et al.* reported that ^68^Ga-FAPI-04 PET/CT demonstrated inferior sensitivity (50.65 *vs.* 96.75%) and accuracy (51.28 *vs.* 95.51%, P < 0.001) than ^18^F-FDG in a subgroup of eight patients with hematological neoplasms, including multiple myeloma and lymphoma 52.

**Figure 2:**
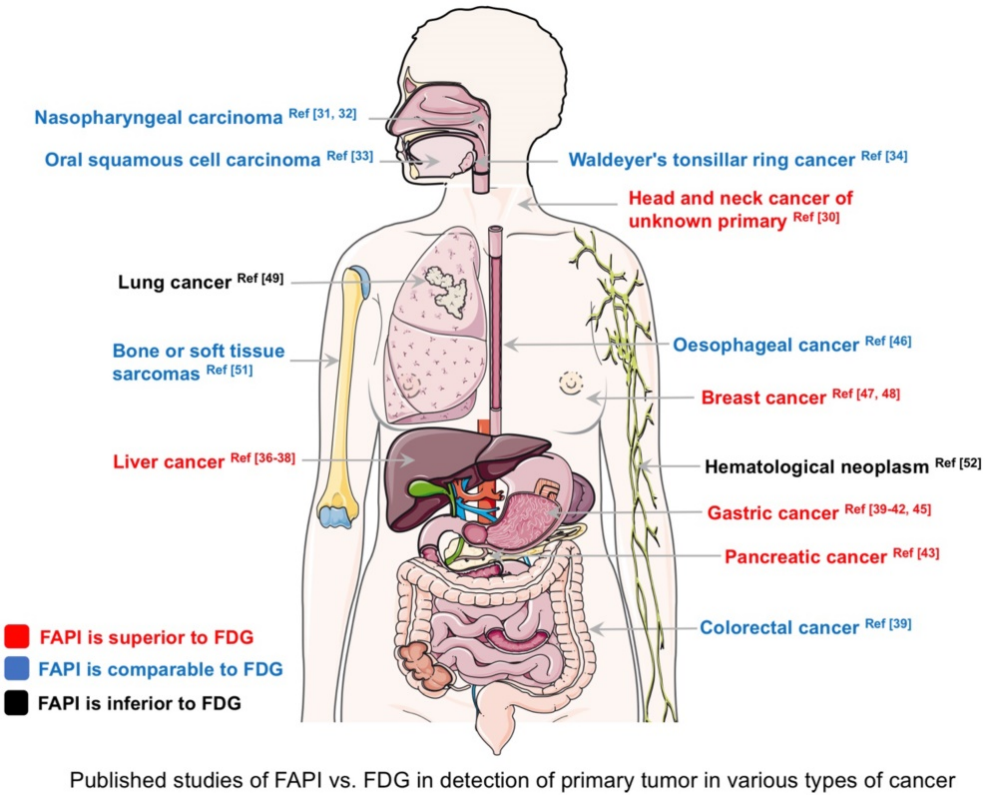
Published studies comparing fibroblast activation protein inhibitor-positron emission tomography (FAPI) *vs.* fluorodeoxyglucose positron emission tomography (FDG) in the diagnosis of various types of cancer (detection rate of primary tumors). The corresponding references are presented in the figure.

The primary lesion detection rates for ^68^Ga-FAPI and ^18^F-FDG PET/CT (based on published data) in various types of cancer are summarized in Figure 2. ^68^Ga-FAPI PET/CT outperformed ^18^F-FDG PET/CT in CUP, breast cancer, hepatocellular carcinoma, intrahepatic cholangiocarcinoma, gastric cancer, and pancreatic cancer. In these diseases, ^68^Ga-FAPI PET/CT may have the potential to replace ^18^F-FDG in the future. Conversely, ^68^Ga-FAPI PET/CT was inferior to ^18^F-FDG PET/CT for diagnosing hematological neoplasms. However, it should be noted that Figure 2 is not based on a systematic literature search of PubMed/MEDLINE and Cochrane Library database analysis, and the number of patients enrolled in previous studies was very small. It should also be noted that the results of Figure 2 only aimed at comparing the detection rate of primary tumors between ^18^F-FDG and FAPI PET/CT based on existing investigations, including retrospective or smaller prospective studies. For more information, a systematic review was conducted by Treglia *et al.* to compare radiolabeled FAPI and ^18^F-FDG PET/CT in oncologic imaging 53. Clear comparability, especially superiority or inferiority, between FAPI *vs*. FDG should be demonstrated in future studies.

Overall, we conclude that ^68^Ga-FAPI PET/CT has comparable or improved diagnostic performance in imaging various cancers compared to 18F-FDG PET/CT, particularly in cancer types that normally show a low-to-moderate ^18^F-FDG uptake 54, 55. Lower background uptake in normal organs leads to higher TBRs with ^68^Ga-FAPI when compared with ^18^F-FDG 56. As stroma components can comprise up to 50-90% of the tumor environment, stroma-targeted PET/CT imaging could be more sensitive than glucose metabolism-based PET imaging in detecting small lesions or lesions with low glucometabolic activity 57. Moreover, ^68^Ga-FAPI PET/CT provides advantages over ^18^F-FDG PET/CT because it does not require fasting and imaging acquisition at early time points following injection (*i.e.*, 10-60 min after tracer administration). However, it is too arbitrary to say FAPI PET/CT will replace FDG PET/CT based on the current clinical studies. The Role of FAPI PET in cancer imaging and management should be further explored in larger prospective trials before any conclusion should be reached.

## Changes in cancer management according to ^68^Ga/^18^F-FAPI PET/CT

**Figure 3:**
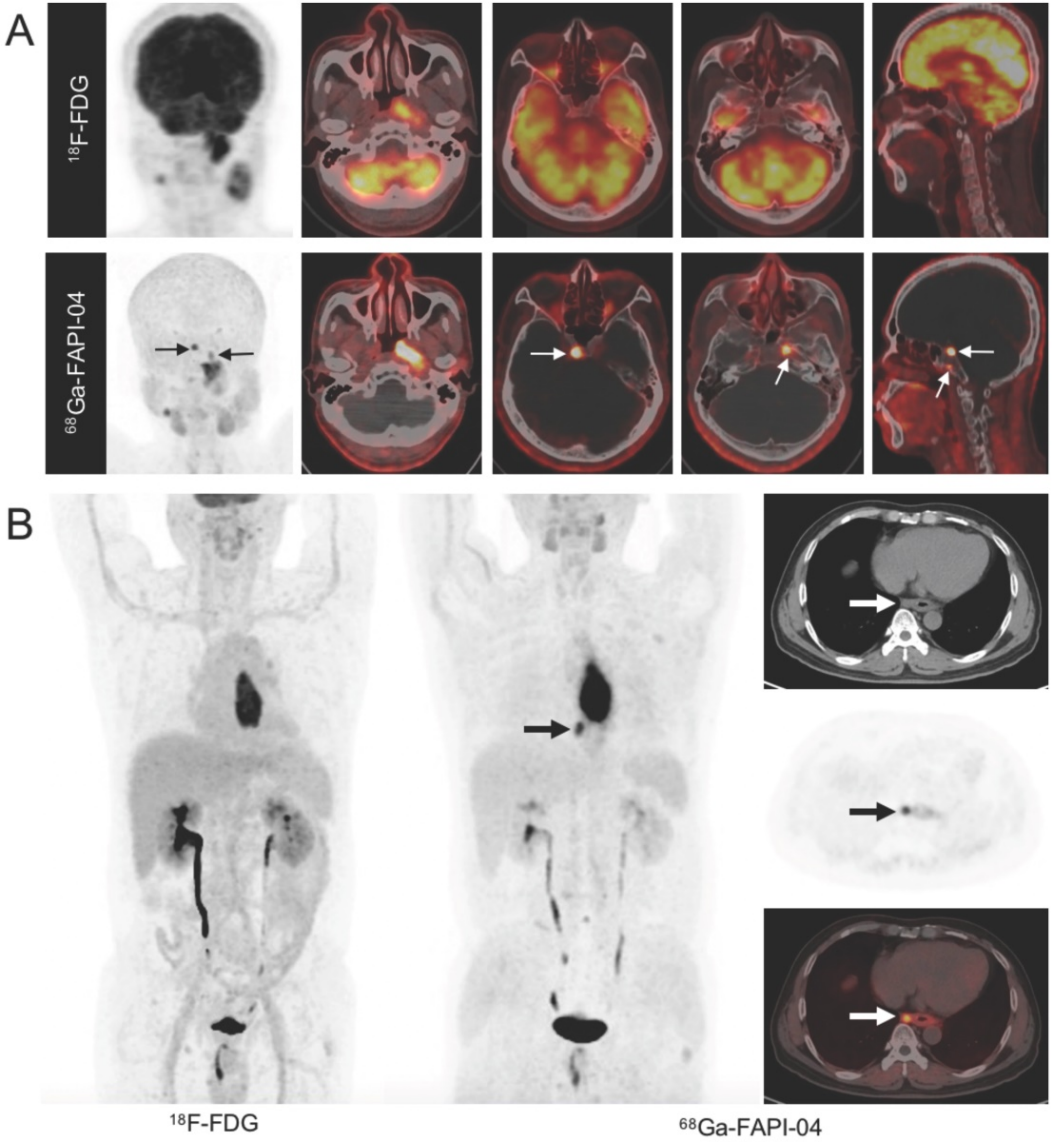
A. Imaging findings in a 51-year-old treatment-naive male patient with nasopharyngeal carcinoma. ^18^F-fluorodeoxyglucose (FDG) (upper row) and ^68^Ga-labeled fibroblast activation protein inhibitor (FAPI) positron emission tomography/computed tomography (PET/CT) (lower row) reveal abnormal activity in the nasopharynx. However, intense ^68^Ga-FAPI uptake is observed in the skull base (white arrow) along with normal FDG uptake, confirmed by magnetic resonance imaging. The TNM stage was upgraded from T2N2 (FDG-based) to T3N2 (FAPI-based). B. Imaging findings in a 56-year-old treatment-naive male patient with esophageal squamous cell carcinoma. ^18^F-FDG PET/CT for tumor staging to decide the most proper treatment strategy. Maximum intensity projection (MIP) image ^18^F-FDG PET/CT reveals an intense FDG-avid mass in the mid-esophagus, while the MIP image of ^68^Ga-FAPI-04 shows intense uptake of FAPI in the primary tumor and paraesophageal lymph node. This FAPI-positive lymph node, suggestive of nodal metastasis, was later confirmed by histopathology. Tumor staging was upgraded to stage IIIB based on FAPI. Adapted with permission from 31, copyright 2021 Springer, and 58, copyright 2020 Springer.

In addition to studies comparing ^68^Ga-FAPI and ^18^F-FDG PET/CT for the diagnosis of various types of cancer, several studies have explored the added value of ^68^Ga-FAPI PET/CT in cancer management compared with standard care of imaging (SCI). In oral squamous cell carcinoma, ^68^Ga-FAPI-04 PET/CT showed superior sensitivity (81.3% *vs.* 50.0%) and specificity (93.3% *vs.* 61.5%) in detecting lymph node metastases compared with CE-MRI 33. Specifically, compared with CE-MRI, ^68^Ga-FAPI PET/CT upgraded and underestimated the T stage in 4/39 and 2/39 patients with NPC, respectively (Figure 3A) 31. Zhao *et al.* reported that ^68^Ga-FAPI-04 PET/CT improved tumor staging in patients with esophageal cancer, compared to contrast-enhanced CT and ^18^F-FDG PET/CT (Figure 3B) 58. Pang *et al.* and Röhrich *et al.* reported that the use of ^68^Ga-FAPI-04/46 PET/CT led to changes in oncologic management in 1/23 patients and 7/19 patients, respectively, due to the upstaging of the TNM stage as compared with contrast-enhanced computed tomography (CE-CT) 43, 44. Regarding anal canal carcinoma, ^68^Ga-FAPI-46 PET/CT led to a change in the TNM classification in 3/6 patients as compared with MRI and CT in a previous study (*i.e.*, based on FAPI-positive nodes in two patients with ill-defined results on MRI and FAPI-negative lesions in one patient with suspected pulmonary lesions on CT) 59.

Optimization of TNM staging *via* FAPI PET/CT results in improving oncologic management. For example, the course of clinical management was changed in 13 (30%) patients with sarcomas following ^68^Ga-FAPI-PET in a previous investigation, including major changes (*e.g.*, changes in therapeutic strategy) in seven (16%) patients 51. However, ^68^Ga-FAPI PET/CT imaging is not always superior to conventional SCI. For example,^ 68^Ga-FAPI-04 PET/CT detected fewer lesions as compared with MRI of the liver (85% [41/ 48] *vs.* 100% [48/48], P = 0.34) in a cohort of 32 patients with liver cancer 36. Therefore, it is recommended that ^68^Ga-FAPI PET/CT be used as a tool complementary to ^18^F-FDG PET/CT and SCI. Additional investigation is required, as current studies investigating the advantages and disadvantages of ^68^Ga-FAPI PET/CT are highly limited.

**Figure 4:**
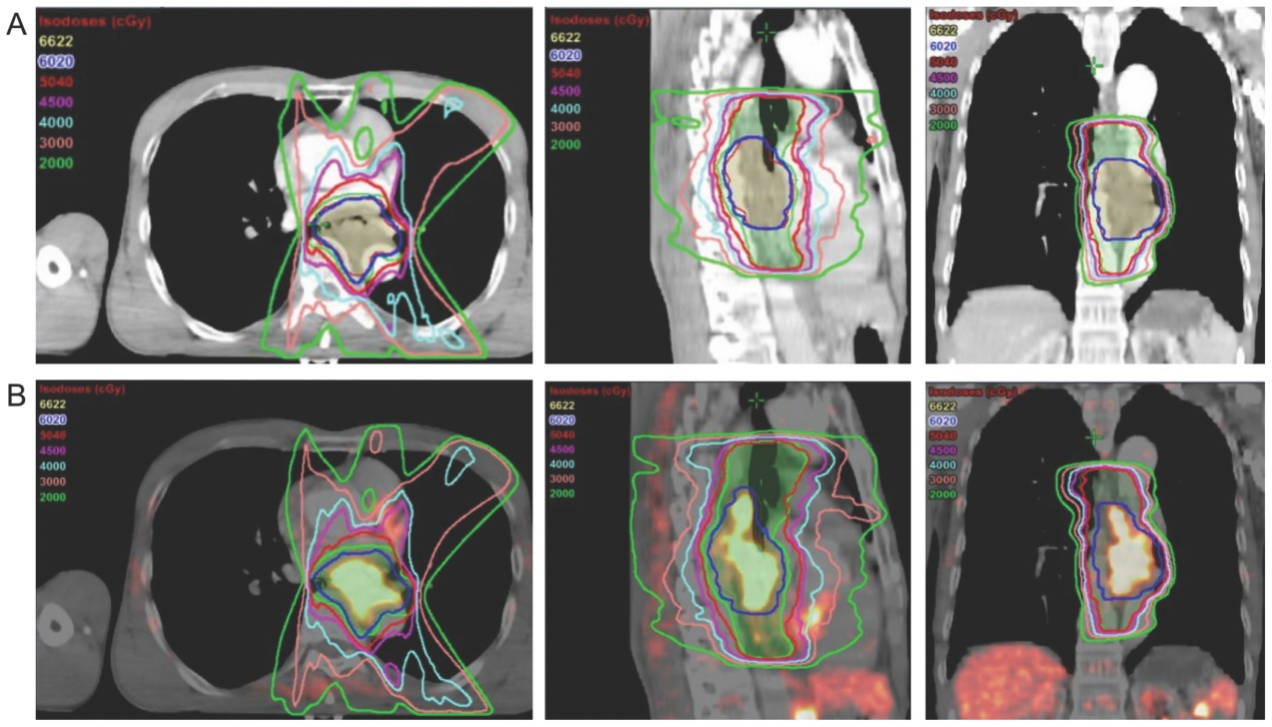
Radiation treatment plan for a 57-year-old male patient with lower esophageal cancer based on (A) contrast-enhanced CT (tumor length, 4 cm; GTV volume, 39.32 cm^3^); and (B) CT + FAPI ×20% (tumor length, 7.5 cm; GTV volume, 41.73 cm^3^). Adapted with permission from 46, copyright 2021 Elsevier.

In addition to changes in TNM classification, a clear contour with a favorable TBR improves target volume delineation for radiotherapy. Syed *et al.* first introduced ^68^Ga-FAPI PET/CT images in tumor radiotherapy for evaluating gross tumor volume (GTV) contouring 60. Four thresholds (three-, five-, seven- and ten times that of the ^68^Ga-FAPI uptake in normal tissue surrounding the tumor) were used to generate FAPI-GTV values (FAPI × 3, 5, 7 and 10) in 14 patients with head and neck cancers, which were subsequently compared with GTV values measured conventionally *via* CE-CT and MRI (CT-GTV). The area covered by FAPI-GTV (FAPI × 3 and 5) was significantly different compared to the area covered by CT-GTV, and the FAPI × 3 threshold was recommended as the best among the imaging modalities 60. Similarly, ^18^F-FAPI-74 × 3 and ^68^Ga-FAPI-04 × 2 thresholds were considered optimal with respect to radiotherapy planning for lung cancer and locally recurrent pancreatic cancer 21, 61. Windisch *et al.* compared FAPI-GTV and MRI-GTV values in glioblastoma and found that the FAPI-GTV values for all thresholds were greater than the MRI-GTV values 62. In addition to using background FAPI uptake, Zhao *et al.* used demarcations at 20%, 30%, and 40% of the SUVmax as thresholds for FAPI-GTV; their results demonstrated that ^68^Ga-FAPI-04 × 40% of the SUVmax was ideal for reflecting the actual tumor volumes in esophageal cancer (Figure 4) 46. Interestingly, PET/CT with different FAPI variants (^68^Ga-FAPI-02, ^68^Ga-FAPI-46, and ^68^Ga-FAPI-74) acquired at three time points (10 min, 1 h, and 3 h) with a threshold of 25-35% of the SUVmax was used to delineate three FAPI-GTVs in adenoid cystic carcinomas, all of which were more accurate than CT-GTVs (based on CE-CT and CE-MRI); the ^68^Ga-FAPI PET/CT images acquired 1 h post-injection were presumed to reflect the ideal time point for contouring 63. Without implementing the FAPI threshold mentioned above, Koerber *et al.* and Ristau *et al.* reported that ^68^Ga-FAPI-04/46 PET/CT improved target volume delineation in 6/6 anal canal carcinoma patients and 6/7 esophageal cancer patients 59, 64.

The published data on the impact of FAPI PET/CT on radiotherapy are shown in Table 1. However, differing FAPI variants and thresholds for various types of tumors may be obstacles to the widespread use of FAPI PET/CT in GTV contouring. A well-designed prospective study with a large patient population is warranted to evaluate the overall survival benefit from FAPI-derived GTVs compared with GTVs derived from SCI in a heterogeneous grouping of cancers and imaging modalities.

## Improvement in the FAPI probe and FAP-targeted radionuclide therapy

As a pan-cancer target with an excellent TBR, FAP is considered an attractive target for radionuclide therapy. FAPI variants labeled with therapeutic radionuclides (such as ^131^I, ^90^Y, ^177^Lu, and ^225^Ac) have been assessed in both preclinical and clinical studies. For example, Ma *et al.* synthesized ^131^I-FAPI-04 and used it to suppress tumor growth in U87MG glioma xenografts 65. FAPI-04 labeled with ^225^Ac demonstrated statistically significant tumor-suppressive effects compared with the control group in a study of pancreatic cancer xenografts 66. Similarly, ^177^Lu-FAPI-46 and ^225^Ac-FAPI-46 showed tumor growth suppression in pancreatic cancer mouse models without an obvious decrease in body weight; no radionuclide therapy-related side effects were observed in these tumor xenografts 67. However, tumor uptake was only 0.3% ID/g at 3 h p.i. and 0.1 % ID/g at 24 h p.i. for ^177^Lu-FAPI-46 and ^225^Ac-FAPI-46 67, respectively, and these results require additional clarification.

**Figure 5:**
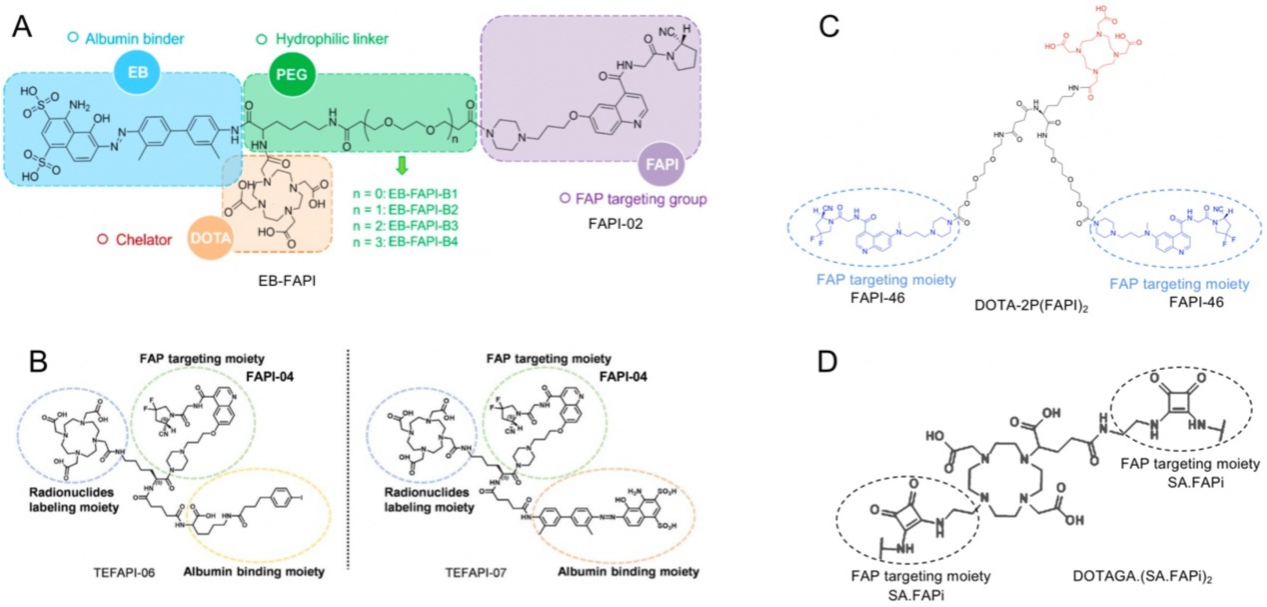
A. Chemical structure and each part of the functional groups of ^177^Lu-EB-FAPI-B1 (without PEG), ^177^Lu-EB-FAPI-B2 (with PEG: n = 1), ^177^Lu-EB-FAPI-B3 (with PEG: n = 2) and ^177^Lu-EB-FAPI-B4 (with PEG: n = 3) (FAP targeting motif: FAPI-02). Adapted with permission from 70, copyright 2021 Ivyspring. B-D. (B) Chemical structure of TEFAPI-06/07 (FAP targeting motif: FAPI-04). Adapted with permission from 71, copyright 2021 Journal of Nuclear Medicine. (C) Chemical structure of DOTA-2P(FAPI)_2_ (FAP targeting motif: FAPI-46). Adapted with permission from 74, copyright 2021 Journal of Nuclear Medicine. (D) Chemical structure of DOTAGA.(SA.FAPi)_2_ (FAP targeting motif: SA.FAPi) Adapted with permission from 83, copyright 2021 Mary Ann Liebert.

Strategies for prolonging the blood circulation of drug molecules by adding albumin-binder moieties and harnessing the polyvalency effects of multimeric peptides are widely used to enhance the tumor uptake and retention of radiopharmaceuticals 68, 69. For example, a series of albumin binder (truncated Evans blue) modified FAPI-02 related radiopharmaceuticals has been synthesized and radiolabeled with ^177^LuCl_3_ (named ^177^Lu-EB-FAPI-B1, B2, B3, B4, Figure 5A). Improved tumor accumulation and retention of these compounds were observed until 96 h post-injection, especially for ^177^Lu-EB-FAPI-B1. ^177^Lu-EB-FAPI-B1 demonstrated notable tumor growth inhibitions in the U87MG tumor model with negligible side effects, indicating that ^177^Lu-EB-FAPI-B1 is a promising theranostic agent for future clinical transformation 70. Similarly, FAPI-04 conjugated with albumin binder (4-[p-iodophenyl] butyric acid moiety, truncated Evans blue moiety, lauric acid [C12], and palmitic acid [C16]) was developed (TEFAPI-06, TEFAPI-07, FAPI-C12, and FAPI-C16) to improve tumor retention (Figure 5B), and novel FAPI-variants showed notable tumor growth inhibition after radiolabeling with ^177^Lu in pancreatic cancer patient-derived xenografts (PDXs) and HT-1080-FAP xenografts 71, 72. Another albumin binder (Lys [4-p-chlorophenyl] butyric acid)-conjugated FAP-targeting peptide (Alb-FAPtp-01) showed higher tumor uptake as compared with FAPI-04 after radiolabelling with ^68^Ga 73. Multimerization has been used as another strategy to improve tumor uptake and retention. Recently, the FAPI dimer DOTA-2P(FAPI)_2_ was synthesized based on the structure of FAPI-46 (Figure 5C), and it demonstrated increased tumor uptake and retention properties compared to FAPI-46 in PDXs of hepatocellular carcinoma 74. Moreover, PET/CT scans in three cancer patients revealed higher intratumoral uptake of ^68^Ga-DOTA-2P(FAPI)2 compared to ^68^Ga-FAPI-46 in 21 tumor lesions (SUVmax: 8.1-39.0 *vs.* 1.7-24.0; P < 0.001) 74.

Regarding the clinical investigation of FAPI-targeted radionuclide therapy, Lindner *et al.* first reported that a patient with advanced breast cancer was treated with 2.9 GBq of ^90^Y-FAPI-04, resulting in a statistically significant reduction in pain medication 17. Other FAPI variants (FAPI-46 and DOTA.SA.FAPi) radiolabeled with therapeutic nuclides (^153^Sm, ^90^Y, and ^177^Lu) were evaluated in several scattered case reports (Table 2) 75, 22, 76-78. Subsequently, a few preliminary studies on FAP-targeted radionuclide treatment were reported.

For example, in a study of 22 cycles of ^177^Lu-FAP-2286 administered to 11 patients with diverse adenocarcinomas (mean injected activity, 5.8 GBq), grade 3 adverse events occurred in three patients, and no grade 4/5 adverse events occurred 79. Ferdinandus *et al.* reported 13 cycles of ^90^Y-FAPI-46 administered to nine patients (with a mean injected activity of 3.8 GBq for the first cycle and a mean injected activity of 7.4 GBq for any subsequent cycle), with new grade 3/4 adverse events occurring in four patients 80. In contrast, only one patient had a new grade 3 adverse event in a study of 36 cycles of ^177^Lu-FAPI-46 administered to 18 patients (median injected activity, 3.7 GBq) 81. The measured mean absorbed dose of ^177^Lu-FAPI-04 was 0.37 Gy/GBq in tumor lesions 82, much lower than that of ^90^Y-FAPI-46 (median, 1.28 Gy/GBq) and ^177^Lu-FAP-2286 (3.00 Gy/GBq in bone metastases) 80, 79. Recently, another FAPI dimer, DOTA(SA.FAPi)_2_, was developed and synthesized by Ballal *et al.* (Figure 5D) 83. Radionuclide therapy with ^177^Lu-DOTA(SA.FAPi)_2_ was administered to 15 patients with radioiodine-refractory differentiated thyroid cancer (DTC; RR-DTC). The results of that study revealed that the numbers of patients with complete response, partial response, and stable disease were 0, 4, and 3, respectively. None of the patients experienced grade 3/4 hematological, renal, or hepato-toxicity.

However, most of the studies mentioned above showed mixed responses to FAP-targeted radionuclide therapy because of the different tumors and patient conditions evaluated (Table 2). It should be noted that the latest studies were mainly aimed at evaluating the feasibility and safety of FAP-targeted radionuclide therapy; the number of patients enrolled in these studies was very limited, and the patient cohorts were heterogeneous. In addition, most patients received FAP-targeted radionuclide therapy as the last line of treatment with poor performance status. Although the tumor half-life of FAP-2286 (average of 44 h for bone and 32 h for single liver metastases) is prolonged compared to FAPI-02/04, it is still shorter than the tumor half-life of PSMA 79, 84.

PSMA-targeted radionuclide therapy is reportedly very effective with beta-radionuclides such as ^177^Lu. Moreover, ^177^Lu-PSMA directly targets cancer cells in radiotherapy, while the ionizing radiation of radiolabeled FAPIs mainly kills CAFs and indirectly kills cancer cells adjacent to CAFs via crossfire effects. These reasons may partially explain the difference in treatment response to targeted radionuclide therapy between PSMA and FAPI. Therefore, further research to enhance the therapeutic efficacy of FAP-targeted radionuclide is of great importance, including optimizing the chemical structure of the FAPI vector (e.g., multimerization and chemical conjugation with albumin binder), shortening the time interval between treatments, increasing the administered dose of therapeutic radionuclide, and combination treatments with other types of treatment (e.g., immunotherapy, external-beam radiotherapy, and molecular targeted therapy).

It has been reported that a tumor size of 1-2 mm requires the formation of stroma to support the tumor 57. Thus, radionuclide therapy targeting FAP may be highly effective for treating advanced cancer patients with widespread metastases. Moreover, in an autochthonous model of pancreatic ductal adenocarcinoma, depleting FAP-positive CAFs induced T-cell accumulation in cancer cells and synergistically enhanced anti-tumor effects within PD-L1 immunotherapy 85. Therefore, exploring optimal combination therapies with radionuclide therapy targeting FAP, especially with respect to immunotherapy, is warranted in future research. It must be noted that FAP is overexpressed in various epithelial cancers and is also expressed in many non-oncological diseases 23. In a cohort of 91 patients, 81.3% of the presenting patients had non-tumor FAPI uptake, including degenerative lesions and physiological uptake in normal salivary glands, mammary glands, and the uterus 86. In order to select patients who are most likely to benefit from this therapeutic regimen, careful pre-therapeutic evaluation with ^68^Ga-FAPI PET/CT is recommended prior to FAP-targeted radionuclide therapy.

## Conclusion

FAPI variants labeled with ^68^Ga or ^18^F have shown impressive results in a broad spectrum of cancers. Well-designed clinical trials with large patient populations are needed to define the role of this diagnostic agent, as ^18^F-FDG is the dominant tracer in clinical oncology at present. In addition to CAFs, intense FAP expression is also related to fibrosis, arthritis, atherosclerosis, and autoimmune diseases. Thus, FAPI uptake in non-malignant diseases must be carefully identified. Regarding FAP-targeted radionuclide therapy, one direction for future research is improving the pharmacokinetic properties of tracers via chemical modification. The other potential direction is to explore optimal combination therapies (*e.g.,* combining with external-beam radiotherapy, chemotherapy, and immunotherapy) to synergistically enhance anti-tumor efficacy. Overall, FAPI-based imaging and therapy of cancer have been a highly vibrant research field over the past few years. We look forward to future studies and rapid translation of the most promising FAPI ligands into the clinical arena to benefit patients with various types of cancer.

## Figures and Tables

**Table 1:** Studies summarizing the impact of fibroblast activation protein inhibitor positron emission tomography/computed tomography (FAPI PET/CT) on the efficacy of radiotherapy.

Study	Patients No.	Tumor type	FAPI variants	Compared imaging modalities	Background	FAPI thresholds	Results / optimal threshold
Windisch *et al.* 62	12	Glioblastoma	^68^Ga-FAPI-02 and ^68^Ga-FAPI-04	CE-MRI	Healthy appearing contralateral brain parenchyma	FAPI × 5, 7, and 10	FAP × 5, × 7 and × 10 increase the MRI-GTV (statistical significance
Syed *et al.* 60	14	Head and neck cancers	^68^Ga-FAPI	CE-CT and MRI	Healthy appearing surrounding tissue	FAPI × 3, 5, 7, and 10	FAPI × 3 (about 20-25% SUVmax)
Röhrich *et al.* 63	12	Adenoid cystic carcinomas	^68^Ga-FAPI-02, ^68^Ga-FAPI-46, and ^68^Ga-FAPI-74	CE-CT and CE-MRI	NA	25-35% of SUVmax at three time points (10 min, 1 h, and 3 h)	The FAPI images acquired 1 h p.i. were considered ideal for contouring
Ristau *et al.* 64	7	Esophageal cancer	^68^Ga-FAPI-04 and ^68^Ga-FAPI-46	Standard CT	Not mentioned	Not mentioned	FAPI PET/CT imaging improved target volume delineation in 6/7 patients
Zhao *et al.* 46	21	Esophageal cancer	^68^Ga-FAPI-04	CE-CT	NA	FAPI × 20%, 30%, and 40% SUVmax	FAPI × 20% SUVmax
Giesel *et al.* 21	10	Lung cancer	^18^F-FAPI-74 and ^68^Ga-FAPI-74	CE-CT	Blood-pool	FAPI × 1.5, 2, 2.5, and 3	FAPI × 3 (about 40-50% SUVmax)
Koerber *et al.* 59	6	Treatment-naïve carcinoma of the anal canal	^68^Ga-FAPI-04 and ^68^Ga-FAPI-46	MRI	Not mentioned	Not mentioned	Modified dose concepts in two patients, improved target volume delineation in six patients
Liermann *et al.* 61	7	Locally recurrent pancreatic cancer	^68^Ga-FAPI-04	CE-CT	Healthy appearing surrounding tissue	FAPI × 1.5, 2, and 2.5	FAPI × 2

CE: contrast-enhanced; CT: computed tomography; MRI: magnetic resonance imaging; NA: not applicable

**Table 2:** Radionuclide therapy targeting fibroblast activation protein (FAP).

Study	Patients No.	Tumor type	FAPI agent	Total cycles	Treatment cycle/ patient	Median injected activity	Response (RECIST)	Treatment-related adverse events in all treatment cycles
Assadi *et al.* 81	18	Ovarian cancer, sarcoma, colon cancer, breast cancer, pancreatic cancer, prostate cancer, cervical cancer, round-cell tumor, lung cancer, anaplastic thyroid cancer, cholangiocarcinoma	^177^Lu-FAPI-46	36	1-4	3.7 GBq (1.85-13.7 GBq)	12 SD, 6 PD	1 patient suffered thrombocytopenia (G1), leukopenia (G1), and anaemia (G3) (CTCAE v4.03)
Ballal *et al.* 83	15	Thyroid Cancer	^177^Lu-DOTAGA.(SA.FAPi)2	45	2-4	8.2 GBq (5.5-14 GBq)	NA	Diarrhoea (G1 in 1 pt) (CTCAE v5.0)
Baum *et al.* 79	11	Pancreatic cancer, breast cancer, ovarian cancer, and rectum cancer	^177^Lu-FAP-2286	22	1-3	5.8 GBq (2.4-9.9 GBq)^a^	2 SD, 9 PD	Hemoglobin (G1 in 2 pts, G2 in 4 pts, and G3 in 1 pt), leukopenia (G2 in 1 pt, and G3 in 2 pts, non-G3 in 2 pts), thrombocytopenia (G3 in 1 pts),^b^ pain flare-up (G3 in 1 pts) (CTCAE v5.0)
Ferdinandus *et al.* 80	9	Sarcoma, pancreatic cancer	^90^Y-FAPI-46	13	1-3	3.8 (3.25-5.40) GBq for the first cycle and 7.4 (7.3-7.5) GBq for any subsequent cycle	4 SD, 4 PD	Hemoglobin (G1 in 2 pts, G2 in 2 pts, and G3 in 4 pts), kidney adverse events (G1 in 1 pt, and G2 in 2 pts), liver adverse events (G1 in 1 pt, G2 in 2 pts, G3 in 1 pt, and G4 in 1 pt), pancreatobiliary adverse events (G1 in 1 pt, G3 in 1 pt, and G4 in 1 pt) (CTCAE v5.0)
Kuyumcu *et al.* 82	4	Breast cancer, thymic carcinoma, thyroid cancer, ovarian carcinosarcoma	^177^Lu-FAPI-04	4	1	0.27 GBq (0.26-0.28 GBq)	NA	NA
Lindner *et al.* 22	2	Ovarian cancer and pancreatic cancer	^90^Y‐FAPI‐46	2	1	6 GBq	NA	NA
Jokar *et al.* 78	1	Breast cancer	^177^Lu-FAPI-46	2	2	3.7 GBq	NA	NA
Rathke *et al.* 77	1	Metachronous metastasized breast cancer and colorectal cancer	^90^Y-FAPI-46	4	4	35.5 GBq	SD for breast cancer and PR for colorectal cancer after 1 cycle, but PD after 4 cycles	NA
Ballal S *et al.* 76	1	Breast cancer	^177^Lu-DOTA.SA.FAPi	1	1	3.2 GBq	Decrease in the intensity of headaches	NA
Lindner *et al.* 17	1	Breast cancer	^90^Y-FAPI-04	1	1	2.9 GBq	Statistically significant reduction in pain medication	NA
Kratochwil *et al.* 75	1	Sarcoma	^153^Sm-FAPI-46 ^90^Y-FAPI-46	3	3	20 GBq for ^153^Sm and 8 GBq for ^90^Y	SD	NA

FAPI: fibroblast activation protein inhibitor; RECIST: Response Evaluation Criteria in Solid Tumors; SD: stable disease; PD: progressive disease; PR: partial response; NA: not applicable; CTCAE: Common Terminology Criteria for Adverse Events. aThis footnote indicates the presentation of means rather than medians. ^b^One patient with hemoglobin (G3), leukopenia (G3), and thrombocytopenia (G3).

## Abbreviations

^18^F-FDG: ^18^F-fluorodeoxyglucose
CAFs: cancer-associated fibroblasts
CCL2: CC-chemokine ligand 2
CE-CT: contrast-enhanced computed tomography
CE-MRI: contrast-enhanced magnetic resonance imaging
CUP: cancers of unknown primary origin
DOTA: dodecane tetraacetic acid
ECM: extracellular matrix
FAP: fibroblast activation protein
FAPIs: FAP inhibitors
GGO: ground-glass opacity
GTV: gross tumor volume
IL-6: interleukin-6
OSCC: oral squamous cell carcinoma
PDGFRβ: platelet-derived growth factor receptor-β
PDXs: patient-derived xenografts
PET: positron emission tomography
p.i.: post injection
SCI: standard care of imaging
TBR: target-to-blood pool ratio
TGFβ: transforming growth factor-β
TME: tumor microenvironment
